# A174 A NOVEL MECHANISM OF CROHN’S DISEASE SEVERITY IN WOMEN: EVALUATING THE IMPACT OF AN ESTROGEN-FARNESOID X RECEPTOR INTERACTION ON INTESTINAL BARRIER FUNCTION

**DOI:** 10.1093/jcag/gwac036.174

**Published:** 2023-03-07

**Authors:** D Taylor, L Donovan, J Choi, R Kim, U Schwarz, A Wilson

**Affiliations:** 1 Department of Physiology & Pharmacology; 2 Schulich School of Medicine & Dentistry; 3 Department of Medicine, Western University, London, Canada

## Abstract

**Background:**

Crohn’s disease (CD) is associated with deficits in intestinal barrier function. Activation of bile acid-sensing nuclear receptor Farnesoid X receptor (*NR1H4*, FXR) is associated with protective effects against reduced intestinal barrier function; namely through promoting tight junction complex genes (TJCGs) and reducing expression of inflammatory cytokines. The *FXR -1G>T* variant is associated with decreased FXR activation and increased risk of, and early progression to, CD-related surgery in females only. It is hypothesized an estrogen-FXR interaction is mediating this effect.

**Purpose:**

We aimed to assess the combined effect of estrogen and FXR genetic variation on intestinal barrier function using a cell-based model and its impact in a clinical cohort.

**Method:**

Caco-2 cells were characterized for expression of TJCGs (zonula occludens-1, occludin, junctional adhesion molecule A, and claudin-1 and claudin-2), FXR, and nuclear estrogen receptors (ERα, ERβ) by qPCR. The influence of FXR activation on TJCGs was characterized by incubation of Caco-2 cells with chenodeoxycholic acid (CDCA). FXR-knockout stable Caco-2 line was developed using CRISPR-Cas9 methods and verified by qPCR and genotyping. The effect of estradiol on expression of TJCGs in FXR-knockout and wildtype Caco-2 cell monolayers was compared by qPCR. Future experiments include comparison of FXR-knockout and wildtype monolayer permeability with estrogen exposure by transwell permeability assay.

The effect of FXR genotype and exogenous estrogen CD severity (surgery, hospitalization, fistulizing disease) was evaluated in our female cohort by multivariate analysis.

**Result(s):**

Increased expression of TJCGs was seen in native Caco-2 monolayers incubated with CDCA. FXR-knockout cell line was then successfully created and confirmed. FXR-knockout cells showed decreased expression of TJCGs with the exception of zonula occludens-1. Estradiol exposure resulted in a dose-dependent decline in TJCGs expression in the wildtype Caco-2 cell line, however this effect was lost in the FXR-knockout cell line.

Preliminary analysis of patient cohort data (n=359) showed exogenous estrogen was associated with lower surgery risk (OR = 0.603, 95% CI= 0.373–0.964, p < 0.05; Fischer’s exact test) and trended towards decreasing fistulizing disease risk in a multiple logistic regression model which included FXR genotype. The association of FXR genotype with increased surgery risk was also confirmed in this logistic regression model.

**Conclusion(s):**

Herein, we show that FXR activity affects expression of TJCGs, and this effect is attenuated by estrogen interactions. Our patient cohort preliminary analysis confirmed an increased CD severity risk associated with FXR genotype and demonstrated a trend of decreasing CD severity with exogenous estrogen exposure. Further studies will assess the mechanisms by which FXR and estrogen interact to influence intestinal permeability.

**Please acknowledge all funding agencies by checking the applicable boxes below:**

Other

**Please indicate your source of funding;:**

Department of Medicine, Western University

**Disclosure of Interest:**

D. Taylor: None Declared, L. Donovan: None Declared, J. Choi: None Declared, R. Kim: None Declared, U. Schwarz: None Declared, A. Wilson Consultant of: Consulting fees from Fresenius Kabi, Speakers bureau of: Speaking fees from Takeda and Pfizer

